# Methotrexate-induced toxidermia and pancytopenia in a patient with ectopic pregnancy: a case report

**DOI:** 10.1186/s13256-021-03111-x

**Published:** 2021-12-07

**Authors:** Valéry Refeno, Naharisoa Giannie Rasamimanana, Baco Abdallah Abasse, Malalafinaritra Patrick Marco Ramarokoto, Mahefaniaina Jean Eustache Fanomezantsoa, Pierana Gabriel Randaoharison

**Affiliations:** 1 Medicine Faculty of Mahajanga, Oncology Department of Professor ZAFISAONA Gabriel Teaching Hospital, Mahajanga, Madagascar; 2 Medicine Faculty of Mahajanga, Emergency and Intensive Care Department of Professor ZAFISAONA Gabriel Teaching Hospital, Mahajanga, Madagascar; 3 Medicine Faculty of Antananarivo, Mother and Child Complex of Professor ZAFISAONA Gabriel Teaching Hospital, Mahajanga, Madagascar; 4 Medicine Faculty of Mahajanga, Mother and Child Complex of Professor ZAFISAONA Gabriel Teaching Hospital, Mahajanga, Madagascar

**Keywords:** Methotrexate, Ectopic pregnancy, Drug eruption, Pancytopenia, Case report

## Abstract

**Background:**

Methotrexate is an anticancer drug from the antimetabolite class. It is also used in gynecology and obstetrics and is the molecule of choice for the medical treatment of ectopic pregnancies. We report a case of toxidermia associated with severe pancytopenia induced by methotrexate for ectopic pregnancy.

**Case presentation:**

A 30-year-old Malagasy (African) woman was admitted to the Emergency and Intensive Care Department for probable toxidermia following injection of 75 mg of methotrexate for an ectopic pregnancy. She had developed generalized erythema, which started 48 hours after the injection. The secondary onset of phlyctenular maculopapular skin lesions, generalized purpura, and erosions of the oral mucosa in a context of febrile jaundice prompted her hospitalization. On admission, the patient presented with febrile neutropenia, pancytopenia, renal failure, and hepatic cytolysis. She received transfusions of fresh whole blood, erythromycin, and amphotericin B. The course was fatal within 2 days of hospitalization. The patient died of multiple organ failure.

**Conclusions:**

Our case is mainly distinguished by the lack of use of granulocyte growth factors and folinic acid. In the event of severe reactions to methotrexate, the management should be multidisciplinary and as much as possible within an intensive care unit.

## Background

Methotrexate (MTX) or 4-amino-10-methyl-folic acid is an anticancer drug from the antimetabolite class. It works by inhibiting dihydrofolate reductase, which is an essential enzyme for the synthesis of purine and pyrimidine bases during cell multiplication. It is commonly used in oncology for both solid cancers and malignant hemopathies [1].

In obstetrics and gynecology, MTX is the treatment of choice for the medical treatment of ectopic pregnancy. It enables results comparable to surgery, which is no longer specifically indicated except in cases of tubal rupture and contraindications to MTX [2]. At low doses, side effects are rarely serious, but when they are they can be life-threatening [1, 2]. We report a case of toxidermia associated with severe pancytopenia induced by MTX for ectopic pregnancy.

## Case presentation

A 30-year-old Malagasy (African) woman, a trader, was admitted to the Emergency and Intensive Care Department of the University Hospital Center Professor ZAFISAONA Gabriel, Mahajanga, Madagascar on 19 May 2020 for probable toxidermia following an intramuscular injection of MTX for ectopic pregnancy. On 5 May 2020, the patient consulted for late menstruation (date of last menstrual period was 25 March 2020). The urine pregnancy test was positive. Pelvic ultrasound showed a gravid anteflexed uterus with a thickened endometrium and a right latero-uterine mass with an anechoic center (11.9 mm) consistent with an ectopic pregnancy of 5 weeks of amenorrhea. She was seen in a gynecological consultation on 7 May 2020. After explaining the diagnosis, the patient accepted medical treatment for the ectopic pregnancy with MTX. The patient received an unique intramuscular injection of MTX at a total dose of 75 mg (1 mg/kg) the same day. There was no immediate incident.

On 9 May 2020 (48 hours after the injection), the patient noticed the appearance of rashes in erythematous patches diffuse all over her body. The evolution was marked by the appearance of blisters and purplish spots. There was secondary onset of fever, nausea, fatigue, and yellowing of the eyes and skin. The appearance of oral ulcers, pain in the throat, and diarrhea in the context of deterioration in general condition motivated the consultation. On 15 May 2020 (eighth day after the injection), neutropenia was reported at 0.5 Giga per liter (G/L), hypercreatininemia at 12 µmol/L, hyponatremia at 132 mmol/L, hypokalemia (3.06 mmol/L), hypochloremia (97 mmol/L), and an increase in C-reactive protein to 152 mg/L. Hemoglobin, platelet, and transaminase levels were normal. After multidisciplinary consultation, the patient was hospitalized in the Emergency and Intensive Care Department on 19 May 2020 for probable toxidermia induced by MTX.

Note that the patient had no medical, familial, or psychosocial history. She had undergone an appendectomy in 2005, and there was no complication. She was known to be allergic to seafood and penicillin.

**Fig. 1:**
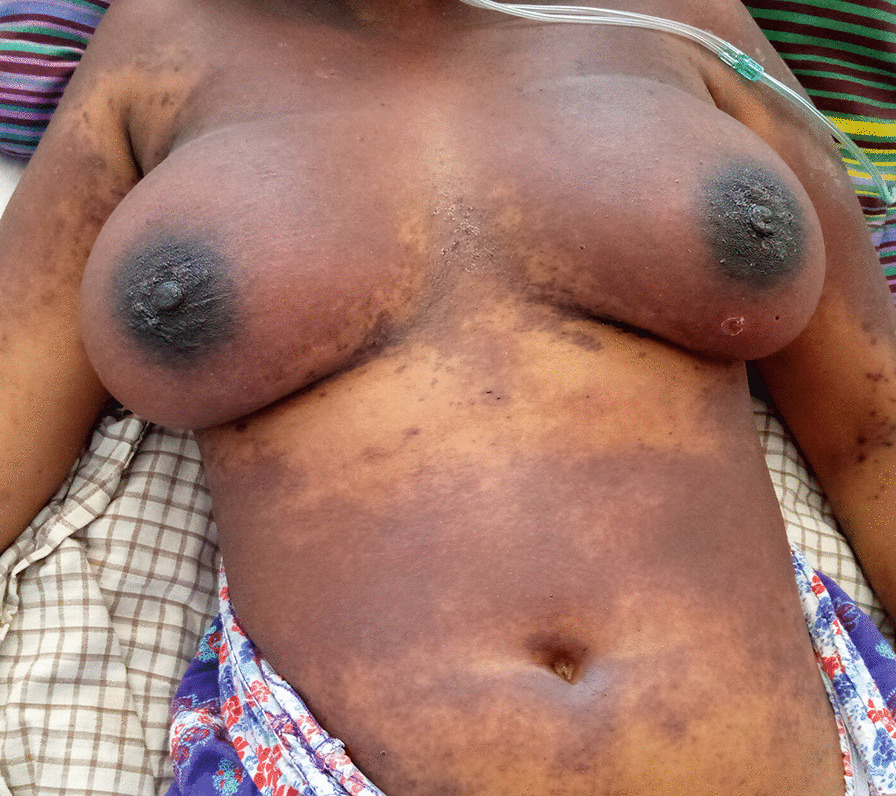
Non-itchy phlyctenular maculopapular eruptions

**Fig. 2:**
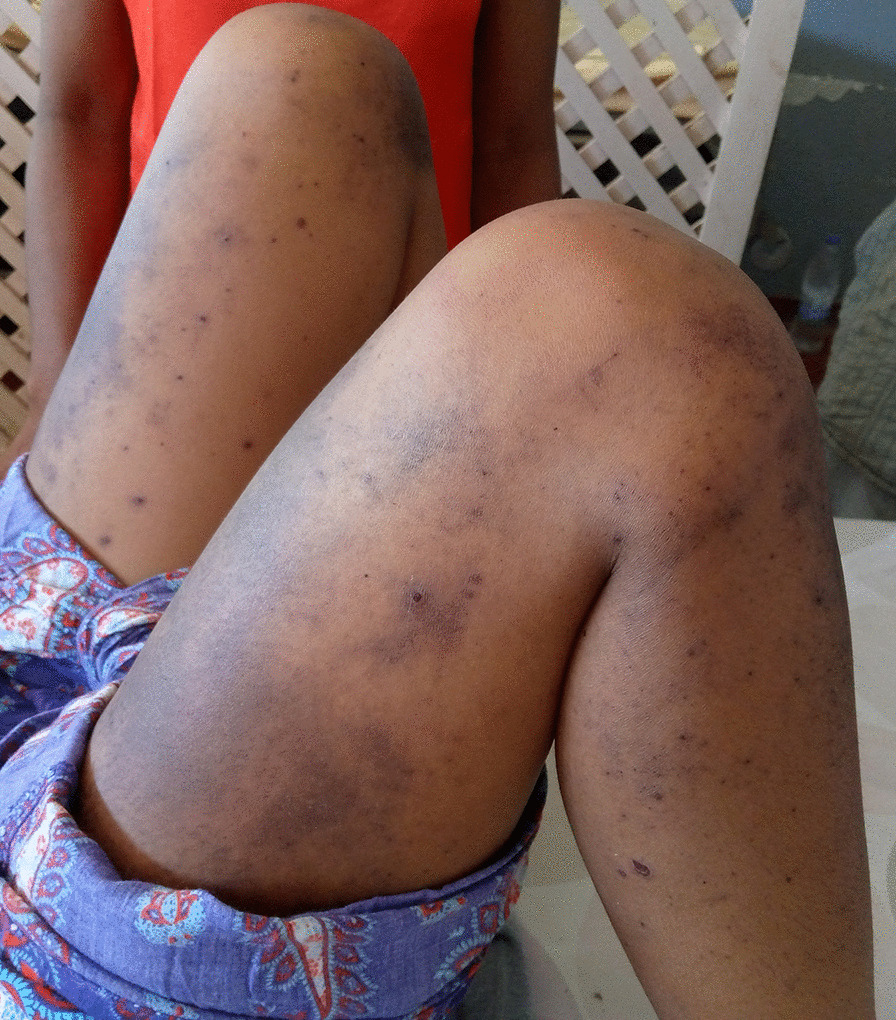
Generalized purpuric purplish lesions

**Fig. 3:**
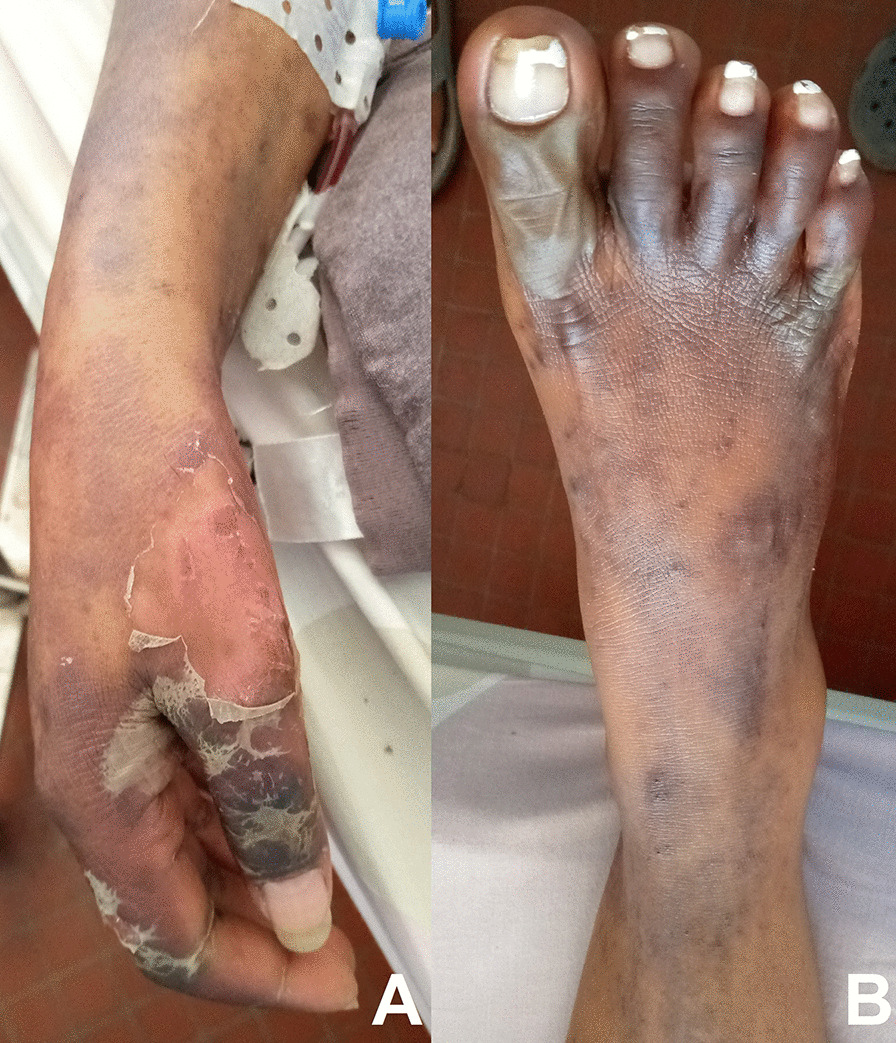
Loss of substance exposing the hypodermis in the hands (**A**) and feet (**B**)

**Fig. 4:**
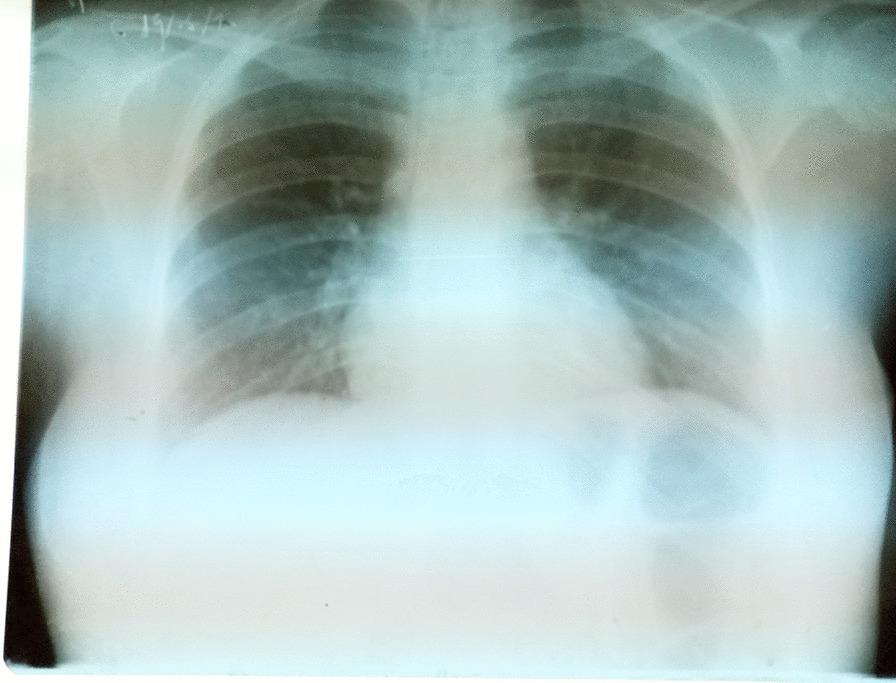
Suspicious interstitial lesions at the level of the left pulmonary bases

On initial examination on 19 May 2020, the patient complained of sialorrhea, odynophagia, and burning of the oral cavity. She was jaundiced, febrile (38.8 °C), dehydrated, and tired. Hemodynamic status was stable. Dermatological examination found nonpruritic phlyctenular maculopapular rashes (Fig. 1) and generalized purpuric purplish lesions (Fig. 2) extending to the face, associated with loss of substance exposing the hypodermis in the extremities (Fig. 3A, B). The patient presented with ulcerations of the lips and labial commissures. Intraoral examination found erosion of the oral and pharyngeal mucosa. Also, palpation of the right hypochondrium was painful. The assessment of 20 May 2020 found anemia (108 g/L), agranulocytosis at 0.1 G/L, and severe thrombocytopenia (32 G/L). There was an elevation of transaminases to 1.5 times normal, a prothrombin level of 78%, an increase in hyponatremia (131 mmol/L), hypokalemia (2.66 mmol/L), and hypochloremia (92 mmol/L). Serum creatinine was 59 µmol/L. Chest x-ray suggested interstitial lesions at the left pulmonary bases (Fig. 4). Serum methotrexate level could not be measured because it is unavailable in Madagascar. The search for *Plasmodium* species by cytology was done systematically because *Plasmodium falciparum*, which causes severe and potentially fatal forms of malaria, is endemic in Madagascar. Neither *Plasmodium falciparum* nor any of the three other endemic *Plasmodium* species (*Plasmodium vivax*, *Plasmodium malariae*, and *Plasmodium ovale*) were found. Serologies for viral hepatitis B and C were requested but were not honored. Acquired immunodeficiency virus serology was not requested due to lack of patient consent. The blood culture did not isolate any pathogen in the blood after 5 days of culture. A new pelvic examination done on 20 May 2020 confirmed the absence of a latero-uterine mass.

The patient benefited from intravenous rehydration, correction of electrolyte disturbances, mouthwashes with chlorhexidine, cutaneous application of fusidic acid 2%, oral loratadine, oral amphotericin B, alkaline drinks, oral erythromycin, intravenous dexamethasone, intravenous omeprazole, and fresh whole blood transfusion. Despite these treatments, the patient’s condition rapidly deteriorated and she presented with dyspnea (oxygen saturation 80%) on 21 May 2020. Subcutaneous filgrastim and intravenous folinic acid were prescribed, but the patient had died on 21 May 2020 (after 2 days of hospitalization) of multiple organ failure before these last treatments could be honored.

## Discussion and conclusions

In the case we are reporting, the time between the injection of MTX and the appearance of the first signs was 2 days. This delay is comparable to that of the cases reported by Lodha *et al.* in India and by Shao *et al.* in Taiwan, which were respectively 2 days and 3 days [3, 4]. This period is shorter than in the cases reported by Toujani *et al.* in Tunisia and that of Soysal in Turkey, which were both 6 days [5, 6]. Note that this delay is longer than that reported by Gupta *et al.* in India for which it was a few hours after injection of MTX [7]. We administered a 75 mg dose of MTX. This dose of MTX is similar to that given in most patients with similar cases [5–7]. Note that Shao *et al.* reported severe reactions in a patient who received 50 mg of MTX [4]. Thus, monitoring of a patient treated with MTX should be systematic even when it is used at low doses, and continue for at least 1 week after its administration. This monitoring should take into account both the efficacy and the tolerance of the treatment.

The patient we are reporting presented with nausea and vomiting as in the cases reported by Shao *et al.* and by Gupta *et al.* [4, 7]. The patient presented with fever, as with the majority of cases reported in the literature [3, 4, 6–8]. The patient presented with generalized phlyctenular maculopapular rashes associated with purpuric lesions and cutaneous jaundice. These skin lesions are the same as those described in the case reported by Gupta *et al.* in India [7]. Skin lesions appear to be polymorphic and vary according to the cases reported. In the case reported by Lodha *et al.*, the patient presented with plate and ulcerated generalized lesions, friable, and bleeding on contact [3]. In the case reported by Soysal *et al.*, the patient presented macular skin rashes localized only in the thorax, neck, and scalp [5]. In the case reported by Toujani *et al.*, the patient presented with generalized erythema with no healthy skin interval associated with ecchymotic patches on the face and limbs [6]. The patient presented with erosion of the oral and pharyngeal mucosa. These mucosal lesions have also been described in the cases reported by Toujani *et al.* in Tunisia and by Lodha *et al.* in India [3, 6]. These mucosal lesions appear to be of varying degrees, ranging from erythema of the oral mucosa to bleeding ulcers of the digestive tract [5, 7].

The patient was anemic on admission as was the case for most of the cases reported in the literature [3, 5, 8]. As in our case, pancytopenia seems constant during severe reactions to MTX [3, 4, 6–8]. Our patient presented with febrile neutropenia as in the cases reported by Shao *et al.* in Taiwan and that of Lodha *et al.* in India [3, 4]. Note that febrile neutropenia is not constant and was absent for the cases reported by Gupta *et al*., Soysal *et al.*, and Jebasingh *et al.* [5, 7, 8]. In our case, there was no renal failure as in the cases reported by Shao *et al.* in Taiwan and by Jebasingh *et al.* in India [4, 8]. Our case differs from those reported by other authors in which treatment with low-dose MTX was complicated by renal failure [3, 6, 7]. The patient presented with hepatic cytolysis as in the cases reported by Toujani *et al.* in Tunisia and by Gupta *et al.* in India [6, 7]. Due to the quality of the chest x-ray, it was not possible to formally confirm the presence of infiltrative lung disease, which was present in the cases reported by Toujani *et al.* as well as Gupta *et al.* [6, 7]. Serum methotrexate level was not measured for our patient as was the case for the majority of reported cases [3, 4, 7]. This is because serum methotrexate level is not available in Madagascar and requires the sending of blood samples abroad. This is generally high for cases for which it could be measured [5, 6]. We remind that the mechanism of toxicity of MTX is not fully understood. Indeed, pancytopenia, digestive intolerance, and stomatitis are explained by the deficiency in folinic acid. The mechanisms are more complex for renal failure, hepatic cytolysis, and pulmonary fibrosis [1].

Regarding management, the patient we are reporting did not benefit from granulocyte growth factors unlike the majority of reported cases [3, 4, 7]. Our case did not benefit from broad-spectrum antibiotics recommended in febrile neutropenia unlike cases reported in the literature [3–6, 8]. Our patient did not receive folinic acid. Folinic acid, which is the “antidote to MTX,” was used routinely in other reported cases even in the absence of serum methotrexate level [3–8]. Our patient did not benefit from a platelet pellet unlike the cases reported by Shao *et al.* and by Soysal *et al.* [4, 5]. Plasmapheresis, which is also an option for rapidly lowering MTX levels, was not used in the patient [5]. Our therapeutic management is explained, on the one hand, by the fact that the platelet pellet and plasmapheresis are not available in Madagascar. On the other hand, the very rapid deterioration of the patient’s condition did not give her family time to buy the granulocyte growth factors and folinic acid, the costs of which remain their responsibility.

The patient's hospital stay (2 days) is short compared with that reported in the literature, which was respectively 4, 14, 14, 18, and 21 days for Toujani *et al.*, Jebasingh *et al.*, Lodha *et al.*, Soysal *et al.*, and Shao *et al.* [3–6, 8]. As in the case reported by Tounaji *et al.* in Tunisia, the patient that we report died of multiple organ failure [6]. This contrasts with the evolution of Asian cases, which was generally favorable with normalization of the blood count and disappearance of skin and mucous lesions [3–5, 7]. Before initiating treatment with MTX, the practitioner must first ensure that there are no absolute and relative contraindications to this treatment [1]. In the event of severe reactions, the management should be multidisciplinary and as much as possible within an intensive care unit.

The case we are reporting differs from the data in the literature by the absence of serum methotrexate level, the absence of the use of granulocytic growth factors, antibiotics for febrile neutropenia, folinic acid, and platelet pellet. It is also distinguished by a very short hospital stay, which resulted in a fatal outcome. In this case, the main problems leading to death of the patient were the lack of broad-spectrum antibiotics for the febrile neutropenia and the lack of folinic acid. The dose and route of MTX were correctly chosen. Neutropenia due to low-dose MTX is rare and can occur after varying times, making it difficult to anticipate. The takeaway main points are that the clinician should always advise the patient of the possibility of these serious side effects, even though they are rare with low-dose MTX. Monitoring of the patient treated with MTX should be systematic even when used at low doses and should be continued for at least 1 week after administration. In the event of severe reactions, the management should be multidisciplinary and as much as possible within an intensive care unit. Emergency supplies of broad-spectrum antibiotics and folinic acid could be lifesaving in the event of similar events. A prospective study on patients treated with MTX would allow us to know the real frequency of side effects and their severity.

## Data Availability

Not applicable.

## Abbreviations

µmol/l: Micromoles per liter
G/L: Giga per liter
mmol/l: Millimoles per liter
MTX: Methotrexate
